# Rare childhood cancers—an increasing entity requiring the need for global consensus and collaboration

**DOI:** 10.1002/cam4.426

**Published:** 2015-02-09

**Authors:** Rishi S Kotecha, Ursula R Kees, Catherine H Cole, Nicholas G Gottardo

**Affiliations:** 1 Department of Haematology and Oncology, Princess Margaret Hospital for ChildrenGPO Box D184, Perth, Western Australia, 6840, Australia; 2 School of Paediatrics and Child Health, University of Western AustraliaGPO Box D184, Perth, Western Australia, 6840, Australia; 3 Telethon Kids Institute, University of Western AustraliaPO Box 855, Perth, Western Australia, 6872, Australia

**Keywords:** Cancer, childhood, infrequent, pediatric, rare

## Abstract

Rare childhood cancers have not benefited to the same extent from the gains that have been made for their frequently occurring counterparts. In recent years, this gap has been recognized and a number of vehicles now exist to improve outcome, including rare tumor groups, disease-specific registries, and clinics. The multitude of approaches has allowed significant progress, however, this framework is limited by patient number and is not inclusive for every type of rare childhood cancer. These shortcomings can be overcome by a single global unified approach to the study of rare childhood tumors.

A rare disease affects a small percentage of the population and the definition differs according to geographical location. In the United States, the Rare Diseases Act of 2002 defines a rare disease as “any disease or condition that affects less than 200,000 persons in the United States,” while the European Commission defines a rare disease as “a life-threatening or chronically debilitating disease with a low prevalence, affecting less than 5 per 10,000 persons in the European Union, and a high level of complexity.”

Using these definitions, childhood cancer as a whole qualifies as a rare disease, with complete prevalence counts of 169,844 for children under 15 years of age in the United States 1. The definitions of a rare disease are based on prevalence as a measure of a chronic condition. However, childhood cancers are subacute illnesses and the burden within a population is better defined using age-specific incidence rates. Even using this more stringent criterion, childhood cancer remains a rare entity with incidence rates of 172.8 per million in the United States, 138.5 per million in Europe, and 157.5 per million in Australia for children aged between 0 and 14 years 1–3. However, despite its rarity, cancer is the most common cause of disease-related death in children from developed countries, second only to unintentional injury for all-cause mortality 2.

Treatment of childhood cancer represents one of the success stories of modern medicine. As recently as the 1950s, children diagnosed with cancer were treated with palliative intent, whereas in the current era a curative approach is employed for the majority of childhood cancers. Since the 1970s, remarkable progress has been made in the understanding, management, and outcome of the more frequently occurring pediatric malignancies 4. In contrast to adults, childhood cancer is classified according to morphology rather than primary site of origin 5. In the developed world, leukemia, central nervous system (CNS) tumors, and lymphoma are the most frequently occurring cancers in children and account for up to 77% of cases, with leukemia comprising 27–35%, CNS tumors 20–27%, and lymphoma 8–15% 3.

The difficulty of performing meaningful studies on the few patients treated in each pediatric oncology center has fostered the establishment of large international cooperative groups, such as the Children's Oncology Group (COG), and scientific organizations, such as the International Society of Paediatric Oncology (SIOP). These groups have facilitated the uniform conduct of clinical trials and molecular epidemiology studies, involving multiple institutions from different countries, enabling accrual of adequate patient numbers, and ultimately leading to significant improvements in outcome for the majority of childhood cancers. Historically, finite resources have directed the focus of these cooperative groups toward improving the outcome of the most frequently occurring childhood cancers, which provide the greatest burden.

However, not all childhood cancers have benefited to the same extent from these advances. This group constitutes tumors so infrequent that they have historically been overlooked due to the limited ability to accrue in a timely fashion onto clinical trials. These barriers have occurred as a consequence of small patient numbers from a heterogeneous group of diseases and the difficulty in establishing funding for rare tumors when competing with the more frequently occurring childhood cancers. Hence, knowledge of the clinical presentation, management, and long-term outcome of these tumors has been based on limited single institution data or extrapolation from adult studies 4.

In recent years, the gap in clinical and scientific knowledge for rare pediatric tumors has been recognized and a number of vehicles now exist to better define and improve outcome for these children. One initiative to study rare childhood tumors has been through the creation of international disease-specific registries and clinics. These include registries for pleuropulmonary blastoma (http://www.ppbregistry.org/), pediatric adrenocortical carcinoma (http://clinicaltrials.gov/show/NCT00700414), NUT midline carcinoma (http://www.nmcregistry.org/), and ovarian and testicular stromal tumors (http://www.otstregistry.org/); and an annual centralized clinic for pediatric gastrointestinal stromal tumor (GIST) (http://www.pediatricgist.cancer.gov/).

Disease-specific international registries provide an opportunity for tumor banking and molecular epidemiology studies. This has led to major scientific discoveries including the identification of a unique *TP53*-R337H germline mutation, which is highly prevalent in Southern Brazil and predisposes to pediatric adrenocortical carcinoma 6; identification of *DICER1* germline mutations in familial pleuropulmonary blastoma 7, which has subsequently led to characterization of the DICER1 syndrome 8; and discovery of *BRD4-NUT-*mediated oncogenic and epigenetic mechanisms in NUT midline carcinoma 9–11. Registries also provide an expert centralized histopathological review to ensure a correct diagnosis is made. This directly benefits patients to ensure that they receive the appropriate therapy and prevents confounding of registry data. Retrospective analysis of collated clinical registry data can better define the epidemiological and prognostic features of the tumor in question and provide an opportunity to make treatment recommendations, as evidenced for adrenocortical carcinoma 12, pleuropulmonary blastoma 13,14, NUT midline carcinoma 15, and prepubertal testis tumors 16.

The development of an annual centralized clinic for pediatric GIST has provided significant benefits for patients, where management is based on a consensus multidisciplinary opinion from international experts in the field. The clinic also facilitates storage of tumor samples, which has enabled the identification of genetic and epigenetic differences between pediatric and adult GIST 17–19.

Another approach has been the development of specialized task forces dedicated to developing clinical and biological research for rare cancers in children, which include the COG Rare Tumors Committee and the SIOP-supported European Cooperative Study Group for Pediatric Rare Tumors (EXPeRT).

The COG Rare Tumor Committee was established in 2002 and initially comprised three subcommittees for germ cell, hepatic, and infrequent tumors. The retinoblastoma subcommittee was incorporated in 2008. Collectively, these four disease categories account for approximately 15–20% of all childhood malignancies 20. The COG has defined infrequent tumors as other malignant epithelial neoplasms and melanomas in the International Classification of Childhood Cancer subgroup XI of the Surveillance Epidemiology and End Results (SEER) database 21. These histologies include adrenocortical carcinoma, thyroid carcinoma, nasopharyngeal carcinoma, malignant melanoma, skin carcinoma, nonmelanoma skin cancers, and other unspecified carcinomas. Although individually these tumors occur infrequently, collectively they account for 9% of all cancers seen in patients younger than 20 years of age in the United States, with three-quarters affecting patients between the ages of 15 and 19 years 21.

Achievements of the COG Rare Tumor Committee include the development of prospective clinical trials for adrenocortical carcinoma and nasopharyngeal carcinoma 20; the development of diagnostic and therapeutic recommendations based on detailed literature reviews for thyroid carcinoma 22, colorectal carcinoma 23, melanoma 24, GIST 25, pancreatoblastoma 26, desmoplastic small round cell tumor 27, gonadal stromal tumor 28, and carcinoid 29; and the design and implementation of a COG group-wide tumor banking study 4.

In Europe, there were a number of national cooperative groups in several countries focusing on the study of rare pediatric tumors prior to 2008. These included the Italian TREP (Tumori Rari in Età Pediatrica) project 30, which was established in 2000; the Rare Tumour Working Group of the United Kingdom's Children's Cancer and Leukaemia Group, which started operating in 1997; the Polish Pediatric Rare Tumor Study Group, established in 2002; the German Pediatric Rare Tumor Working group 31, created in 2006; and the French rare tumor group, FRACTURE (groupe FRAnCais des TUmeurs Rares de l'Enfant), formed in 2007. These groups cooperated to found EXPeRT in 2008, allowing a unified approach to the study of very rare pediatric tumors in Europe. Very rare tumors have been defined by EXPeRT as any solid malignancy or borderline tumor characterized by an annual incidence of less than two per million and/or not already considered in clinical trials 32. This definition incorporates the characterization of very rare tumors as orphan diseases, which by definition indicates that neither clinical nor scientific structures have been developed to aid in their diagnosis or treatment 32. EXPeRT has been successful in uniting a number of European national working groups for rare childhood tumors and has published combined registry data for pancreatoblastoma 33, pleuropulmonary blastoma 34, and ovarian Sertoli–Leydig cell tumors 35.

The multitude of approaches into the study of rare childhood tumors has yielded remarkable clinical and biological success for a number of rare childhood cancers; however, for each individual tumor type significant resources are required to make such advances. Due to the broad heterogeneity of tumor types encompassing rare childhood tumors, the limited resources of the different groups, registries, and clinics has resulted in disparity within the clinical and biological study of rare pediatric tumors. While there is appropriate representation for certain tumor types or subtypes such as pleuropulmonary blastoma, adrenocortical carcinoma, and nasopharyngeal carcinoma, there is a paucity of clinical and/or biological representation for the majority of the other rare childhood tumors.

In addition, the lack of a single unified approach has identified a number of limitations. The definition of infrequent tumors by the COG and very rare tumors by EXPeRT has attempted to capture the majority of those tumors considered rare within the pediatric setting. However, the definitions are nonsynonymous and neither is fully comprehensive, with the consequent exclusion of certain rare childhood tumors by each group. Infrequent tumors as defined by the COG result in the exclusion of tumors such as pleuropulmonary blastoma, pancreatoblastoma, sex cord stromal tumors 32, and GIST, whereas very rare tumors as defined by EXPeRT result in the exclusion of thyroid carcinoma and melanoma, whose incidence rates are 4.9 and 4.6 per million, respectively, in children younger than 20 years of age 4.

Focused initiatives coordinated by disease-specific registries have been major drivers for research into rare childhood cancers; however, they are limited in their ability to perform prospective studies. In addition, the divided approach between disease-specific registries and rare tumor groups has resulted in competing interests for certain conditions such as pleuropulmonary blastoma 13,14,34. It is feasible to have competing interests for frequently occurring childhood cancers as studies are not limited by patient numbers, and the use of different approaches for frequently occurring childhood cancers is ultimately beneficial, as it allows for selection of studies that achieve the best outcome upon which to build future clinical and biological research. However, such an approach is impractical for the study of rare childhood tumors, as the low incidence prevents accrual onto studies in a timely fashion and as a consequence, final outcome measures are often limited in their findings.

Such difficulties have now become applicable to some of the more frequently occurring childhood cancers, due to the discovery of molecular subgroups within tumor types 36,37. Such challenges can be highlighted by medulloblastoma, which has been classified into four distinct molecularly defined subgroups, namely Wnt/Wingless (WNT), Sonic Hedgehog (SHH), Group 3, and Group 4, which have different prognostic and therapeutic considerations. Categorization into molecularly defined subgroups requiring different therapeutic strategies has resulted in the need for subgroup-specific clinical trials (http://clinicaltrials.gov/show/NCT01878617). Recent evidence has shown that even within these four classic subgroups, there is evidence for further subcategorization. This includes the use of cytogenetic biomarkers to stratify SHH, Group 3, and Group 4 tumors into clinical risk groups 38, and stratification of SHH medulloblastoma according to the underlying mutation to predict clinical response to smoothened inhibition 39. Molecular subgrouping results in a dramatic reduction in the number of patients from which to recruit onto clinical trials and molecular epidemiology studies and they consequently face the same difficulties as encountered by rare childhood tumors.

These shortcomings can be overcome by a single global unified approach to the study of rare childhood tumors and subgroups of the more frequently occurring childhood cancers. Although inherent administrative, financial, legal, drug supply, and regulatory difficulties may hinder the establishment of single unified studies for each rare childhood tumor, they are not insurmountable. Examples include the conduct of several global collaborative prospective pediatric cancer trials, including the European and American Osteosarcoma Study Group (EURAMOS) trial and the Intergroup trial for B-cell Non-Hodgkin lymphoma/mature B-cell leukemia; and the analysis of pooled clinical data to determine disease-specific prognostic and therapeutic features, highlighted by initiatives of the Children's Hepatic tumor International Collaboration (CHIC) 20, the Malignant Germ Cell Tumor International Collaborative (MaGIC) 40, and for child and adolescent meningioma 41. To facilitate such an approach, an international working group for rare childhood tumors should initially be established, followed by a consensus meeting whereby a unified definition for rare childhood tumors and strategies for future collaborative research are proposed, including the development of virtual tumor banks to enhance translational research, as have been successfully established for rare adult tumors such as mesothelioma (http://www.mesotissue.org/). The success of establishing an international collaborative group to achieve global consensus can be exemplified by the proceedings of the International Medulloblastoma Working Group 42, the International Ponte di Legno Working Group for acute lymphoblastic leukemia 43, and the International Retinoblastoma Staging Working Group (IRSWG) 44. Development of such a concerted international approach is a necessity to achieve the same magnitude of gains for rare childhood tumors, as has been seen for their more frequently occurring counterparts.

## Conflict of Interest

None declared.
